# ASO Author Reflections: Temporal Trends in Urinary Diversion in Radical Cystectomies from Europe’s Largest Monocentric Cohort with 2224 Cases Over 36 Years

**DOI:** 10.1245/s10434-024-15858-w

**Published:** 2024-07-21

**Authors:** Gregor Duwe, Maximilian Peter Brandt, Axel Haferkamp

**Affiliations:** Department of Urology and Pediatric Urology, University Medical Center of the Johannes Gutenberg-University Mainz, Mainz, Germany

## Past

Radical cystectomy (RC) is one of the most complex operative procedures in urology, and choice of urinary diversion (UD) has undergone significant changes over the last decades. While several nationwide analyses revealed overall higher rates of continent UD (CUD) in Germany as compared with the United States (U.S.),^1,2^ recent large U.S. high-volume centers reported higher rates of CUD in academic high-volume centers compared with smaller hospitals in the U.S. as well as in European cohorts.^3,4^ Over the past four decades, various CUD and incontinent UD (IUD) techniques have been applied at our department, and the ‘Mainz Pouch’ (MP-I and -II) was developed, resulting in one of the largest centers for RC in Germany to date.^5^ Thus, we sought to evaluate trends in UD over a 36-year time period with historical and contemporary patient groups undergoing RC at our department.

## Present

In total, we identified 2224 patients (77% male and 33% female) who underwent RC in our department from 1986 to 2022.^6^ First, we observed a significant increase in mean age from approximately 60 years (1986–1990) to 70 years (2016–2022). Notably, we described a significant increase in patients ≥80 years of age, from 1% (1986–1990) towards 17% (2016–2022). Next, we identified 776 (35%) patients who received CUD compared with 1406 (63%) patients with IUD. Over time, the proportion of CUDs gradually declined from 44% (1986–1990) to 18% (2016–2022). The most commonly used CUDs were the (heterotopic and orthotopic) MP-I with 610 (27%) patients, the MP-II with 80 (3.6%) patients, and the ileal neobladder with 68 (3.1%) patients. Finally, patients who were male, younger, and had no hydronephrosis prior to RC were more likely to receive CUD. The latest developments were characterized by the start of robot-assisted RC in 2016. While 15 robotic-assisted RCs were performed between 2016 and 2020, the proportion increased towards 42 (31.6% related to 133 RCs in total) robot-assisted RCs in 2021 and 2022.

## Future

We present the largest European single-center cohort of UD after RC. Over the 36-year period, our data reflect a significant temporal shift away from CUD to IUD, accompanied by an increase in patients’ age, which might be explained by a demographic change. Moreover, our data mirror the development and extensive experience with the MP-I in the 1980s and 1990s. Our key results of increasing patient age and decreasing choice of CUD are comparable with high-volume U.S. cohorts.^3,4^ In summary, our results underline historical differences in choice of UD between the U.S. and Germany that are increasingly converging, while a center-related heterogeneity remains. In future, we expect a further increase in robotic-assisted RC.
